# Endotracheal tube biofilm translocation in the lateral Trendelenburg position

**DOI:** 10.1186/s13054-015-0785-0

**Published:** 2015-02-27

**Authors:** Gianluigi Li Bassi, Laia Fernandez-Barat, Lina Saucedo, Valeria Giunta, Joan Daniel Marti, Otavio Tavares Ranzani, Eli Aguilera Xiol, Montserrat Rigol, Ignasi Roca, Laura Muñoz, Nestor Luque, Mariano Esperatti, Maria Adela Saco, Jose Ramirez, Jordi Vila, Miguel Ferrer, Antoni Torres

**Affiliations:** Pulmonary and Critical Care Unit, Hospital Clínic, Calle Villarroel 170, Esc 6/8 Planta 2, 08036 Barcelona, Spain; Institut d’Investigacions Biomèdiques August Pi i Sunyer (IDIBAPS), Barcelona, Spain; Centro de Investigación Biomedica En Red- Enfermedades Respiratorias (CIBERES), Mallorca, Spain; University of Milan, Milan, Italy; Hospital das Clínicas, Faculdade de Medicina da Universidade de São Paulo, Pulmonary Intensive Care Unit, São Paulo, Brazil; Department of Clinical Microbiology, School of Medicine, and Barcelona Centre for International Health Research, (CRESIB) Hospital Clínic, Universitat de Barcelona, Barcelona, Spain; Pathology Department, Hospital Clinic, Barcelona, Spain; University of Barcelona, Barcelona, Spain

## Abstract

**Introduction:**

Laboratory studies demonstrated that the lateral Trendelenburg position (LTP) is superior to the semirecumbent position (SRP) in the prevention of ventilator-associated pulmonary infections. We assessed whether the LTP could also prevent pulmonary colonization and infections caused by an endotracheal tube (ETT) biofilm.

**Methods:**

Eighteen pigs were intubated with ETTs colonized by *Pseudomonas aeruginosa* biofilm. Pigs were positioned in LTP and randomized to be on mechanical ventilatin (MV) up to 24 hour, 48 hour, 48 hour with acute lung injury (ALI) by oleic acid and 72 hour. Bacteriologic and microscopy studies confirmed presence of biofilm within the ETT. Upon autopsy, samples from the proximal and distal airways were excised for *P.aeruginosa* quantification. Ventilator-associated tracheobronchitis (VAT) was confirmed by bronchial tissue culture ≥3 log colony forming units per gram (cfu/g). In pulmonary lobes with gross findings of pneumonia, ventilator-associated pneumonia (VAP) was confirmed by lung tissue culture ≥3 log cfu/g.

**Results:**

*P.aeruginosa* colonized the internal lumen of 16 out of 18 ETTs (88.89%), and a mature biofilm was consistently present. *P.aeruginosa* colonization did not differ among groups, and was found in 23.6% of samples from the proximal airways, and in 7.1% from the distal bronchi (*P* = 0.001). Animals of the 24 hour group never developed respiratory infections, whereas 20%, 60% and 25% of the animals in group 48 hour, 48 hour-ALI and 72 hour developed *P.aeruginosa* VAT, respectively (*P* = 0.327). Nevertheless, VAP never developed.

**Conclusions:**

Our findings imply that during the course of invasive MV up to 72 hour, an ETT *P.aeruginosa* biofilm hastily colonizes the respiratory tract. Yet, the LTP compartmentalizes colonization and infection within the proximal airways and VAP never develops.

## Introduction

Critically ill, intubated patients are commonly kept in the semirecumbent position to prevent the gastro-pulmonary route of endogenous colonization [1] and ventilator-associated pneumonia (VAP) [2]. In recent years, consistent data from laboratory studies [3-5] have suggested that the lateral Trendelenburg position (LTP) might be superior to the semirecumbent position in the prevention of VAP. In the LTP, the trachea is oriented below horizontal; thus, aspiration of bacteria-laden oropharyngeal secretions across the endotracheal tube (ETT) cuff is averted [4,5]. Additionally, in such a position the mucus clearance rate is strongly enhanced [3,6]. Currently, a multicenter clinical trial is testing in critically ill patients the efficacy, safety and feasibility of the LTP in the prevention of VAP (available from ClinicalTrials.gov, NLM Identifier: NCT01138540).

Nevertheless, during invasive mechanical ventilation (MV), aspiration of oropharyngeal pathogens is not the only pathogenic mechanism for the development of VAP. In particular, the ETT is commonly made of polyvinylchloride and some bacterial species avidly adhere to polyvinylchloride and form biofilm [7,8]. Bacteria organized into this defensive milieu are shielded by antibiotics [9] and circumvent the host immune response [10]. ETT biofilm has been consistently associated with VAP [11-14]. Indeed, in the majority of patients who developed VAP, Adair *et al*. [13] found a strict association between pathogens cultured from the ETT biofilm and the lower respiratory tract. More recently Wilson *et a*l. [15] found that mature biofilm (stage III/IV) was associated with the development of VAP.

Importantly, the role of the LTP in the prevention of pulmonary colonization and infections caused by ETT biofilm is yet to be investigated. Thus, we set out to examine the dynamics of pulmonary colonization by ETT *Pseudomonas aeruginosa* biofilm in animals positioned in the LTP. In particular, we evaluated whether the time on MV increased the risk of bronchial colonization and pulmonary infections, and whether translocation of pathogens from ETT biofilm and the development of pulmonary infections could be facilitated by concomitant lung injury.

## Materials and methods

The Institutional Ethics Committee approved the protocol, as fully detailed in the acknowledgment section. Animals were managed according to the National Institutes of Health guidelines for the use and care of animals [16].

### ETT *P. aeruginosa* biofilm

ETTs colonized by *P. aeruginosa* biofilm were obtained from a previous study [5]. During this associated study, animals were challenged twice with 5 mL of 10^7^ to 10^8^ colony forming units (cfu)/mL of a log-phase culture of *P.aeruginosa* into the oropharynx. We employed a respiratory isolate of ceftriaxone-resistant, biofilm-producer *P.aeruginosa*, derived from *P.aeruginosa* ATCC 27853. Following bacterial challenge, pigs were ventilated for 66 hours to allow substantial colonization of the ETT by *P.aeruginosa,* and biofilm formation [17]. Prior to the autopsy, secretions within the ETT were aspirated and plated on MacConkey media to identify Gram-negative aerobic bacteria and quantification of *P.aeruginosa*. Upon extubation, the ETT was placed in a sealed specimen bag and stored at −80°C. Finally, prior to its use in the current study, the ETT was stored during a 24-hour period at −4°C for slow thawing.

### Animal preparation and handling

Eighteen pigs (weight 31.5 ± 2.12 Kg; range 29 to 36 Kg) were induced [18], intubated with the aforementioned ETTs and connected to a mechanical ventilator (SERVO-I, Maquet, Fairfield, NJ USA). Internal ETT cuff pressure was maintained at 28 cm H_2_0 through a mechanical device [19]. Anesthesia was maintained with a continuous infusion of midazolam, 0.2 to 0.8 mg/Kg/h, and fentanyl 5 to 10 μg/Kg/h, in order to maintain cessation of spontaneous movements, following painful stimulation. Pigs were ventilated in volume-control, square-wave inspiratory flow, inspiratory fraction of oxygen of 40%, duty cycle of 0.25, tidal volume 8 mL/Kg, without positive end-expiratory pressure (PEEP), and respiratory rate adjusted to maintain Pa_CO2_ within the physiologic range. Inspiratory gases were conditioned through a heated humidifier (Conchatherm III, Hudson RCI, Temecula, CA, USA). Throughout the study, 50 mg/Kg of ceftriaxone was administered every 12 hours to prevent pulmonary colonization by endogenous pathogens. Endotracheal suctioning was performed every 6 hours or when clinically indicated. Importantly, saline was never instilled into the airways throughout the suction procedure. Quality of mucus (normal or purulent) was recorded. Body temperature and white blood cells count were assessed every 12 hours.

### Randomization

Following surgical preparation, pigs were placed in the lateral position with a slight Trendelenburg (−5°). Then, pigs were randomized into the following groups: 1) group 24 h: on MV for 24 hours; 2) group 48 h: on MV for 48 hours; 3) group 48 h/acute lung injury (ALI): in an additional group on MV for 48 hours, ALI was induced as previously reported [20]. Briefly, 0.08 ml/Kg of oleic acid (Sigma-Aldrich, St Louis, MO, USA) emulsified into 18 mL of blood was slowly injected into the right atrium. To produce uniform injury, the total dose of oleic acid-blood solution was partitioned into three equal aliquot portions, which were sequentially injected into the distal port of the central venous catheter with the pig in three positions: prone, right lateral, and left lateral; group 72 h: on MV for 72 hours.

### Respiratory measurements

Ventilatory settings were evaluated every 6 hours and adjusted to maintain blood gases within the physiologic range. Respiratory mechanics were measured daily, as previously reported [21]. Following the assessment of pulmonary variables, arterial gas exchanges were evaluated.

### Autopsy, microbiological studies and ventilator-associated tracheobronchitis (VAT)/VAP definitions

**Figure 1:**
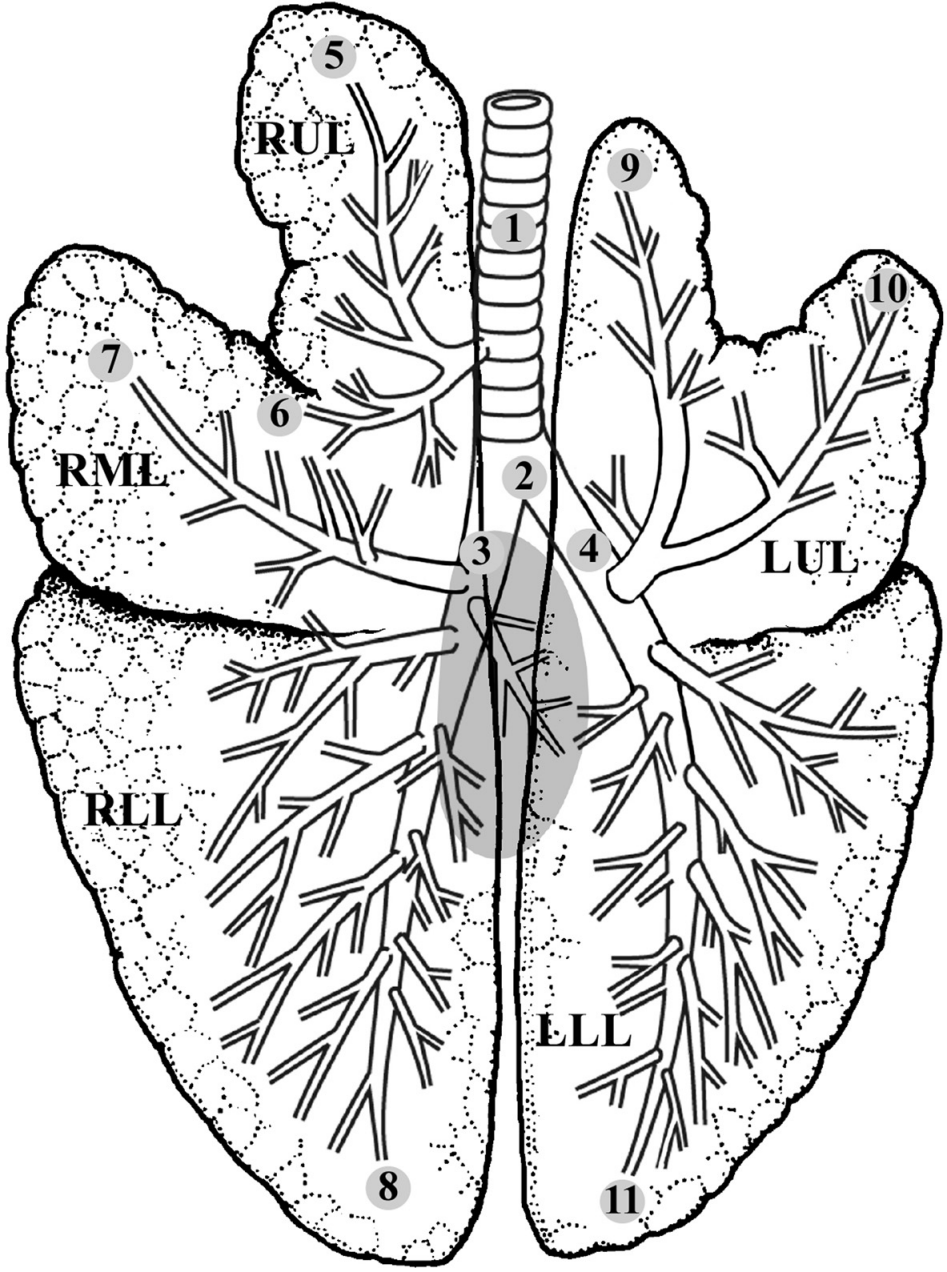
**Bronchopulmonary anatomy of the pig lung.** The numbers from 1 to 11 indicate sites from which samples were taken for microbiological studies: 1 to 2, trachea; 3 to 4, main bronchi; 5 to 11, segmental bronchi. RUL, right upper lobe; RML, right medium lobe; RLL, right lower lobe; LUL, left upper lobe; LLL, left lower lobe.

Upon autopsy, we took four tissue samples from the proximal airways and seven samples from segmental bronchi (Figure 1). Tissue samples were plated on MacConkey agar and incubated aerobically for 48 hours at 37°C. Finally, bacterial growth was counted and pathogens identified by mass spectrometry through a Microflex LT (BrukerDaltonics GmbH, Leipzig, Germany), and bacterial identification was performed using the MALDI BioTyper 2.0 software (BrukerDaltonics). This system measures highly abundant bacterial proteins. The characteristic patterns of these proteins are used to identify a particular microorganism by matching the respective protein spectrum with an extensive database [22]. Pulmonary infections were clinically suspected in case at least two of the following clinical features were present at the end of the study: body temperature >38.5°C or <36°C; white blood count >14,000/mm^3^ or <4000/mm^3^ and purulent secretions. VAT was microbiologically confirmed when at least one bronchial tissue culture was ≥3 log cfu/g. Additionally upon autopsy pulmonary lobes with any apparent gross finding of pneumonia (edematous tissue, areas of consolidation, mucopurulent material in the tracheobronchial tree, abscess) were sampled. Importantly, in group 48-ALI, all lobes were sampled due to the extensive pulmonary injury, irrespective of gross findings of pneumonia. VAP was microbiologically confirmed according to a quantitative lobar bacterial culture ≥3 log cfu/g [23,24].

### ETT microbiological studies

Upon extubation, the ETT was sealed with clamps placed at the proximal and distal tips. The external surface was cleaned with sterile gauzes and decontaminated through careful rinsing with 80% alcohol and saline solutions. Then, the ETT was longitudinally cut open, and a 3-cm-long section of the dependent half was dissected to quantify Gram-negative and Gram-positive aerobic pathogens as previously reported [25]. In two animals of each study group, two 1-cm-long hemi-sections of the ETT were dissected to visually confirm presence of ETT biofilm through confocal scanning laser microscopy (CSLM) [26] and scanning electron microscopy (SEM) [25]. Biofilm stage was assessed through SEM micrographs analysis, as previously described [15].

### Statistical analysis

To the best of our knowledge, only one study [17] in dogs that were challenged with *P.aeruginosa* in the oropharynx has assessed the role of ETT biofilm on pulmonary colonization. Based on these limited data, we assumed a mean *P.aeruginosa* bronchial burden in group 24 h, 48 h, 48-ALI and 72 h of 2, 3, 5 and 4 log cfu/g, respectively, and a standard deviation of 0.5 log cfu/g for each group. Thus, we calculated that approximately three animals should have been included in each group to detect statistically significant differences for a statistical power of 90% and type 1 bias of 5%. Parametric and nonparametric analyses were used in accordance with the results of the Shapiro-Wilk *W*-test. One-way analysis of variance (ANOVA) or the Kruskal-Wallis test with post-hoc Student’s *t*-test or Wilcoxon Mann–Whitney test and Bonferroni correction, was employed to analyze continuous variables. The paired *t*-test and Wilcoxon signed ranks test were used to detect differences between paired measurements. Categorical variables were analyzed using the chi-square and Fisher’s exact test. Of note, only animals with ETTs colonized by *P.aeruginosa* were included in the analysis of tracheobronchial colonization by *P.aeruginosa.* In post-hoc analyses, linear regression and the Wilcoxon Mann–Whitney tests were used to assess the effects of length of stay on MV and lung injury on *P.aeruginosa* lung burden. A two-sided *P*-value <0.05 was considered statistically significant. All analyses were performed using SAS 9.2 software.

## Results

### Study animals

Four animals were included in the 24 h and 72 h group, whereas, five were included in the 48 h and 48 h/ALI groups. Seventeen out of eighteen animals completed the study, one animal in the 48 h-ALI group died after 18 hours due to severe respiratory instability.

### Endotracheal tube colonization

Prior to the current study, ETTs were frozen for 60.7 ± 21.5 days in group 24 h; 47.6 ± 41.3 in group 48 h; 44.8 ± 33.4 in group 48 h-ALI and 58.2 ± 38.9 in group 72 h (*P* = 0.879). Upon completion of the previous study, the secretions collected from the ETT inner lumen in groups 24 h, 48 h and 72 h were always colonized by *P.aeruginosa* at a mean concentration of 5.89 ± 0.95 log cfu/mL without differences among groups (*P* = 0.983). Amid other Gram-negative pathogens, secretions were colonized by *Escherichia coli* in three ETTs (13.04%), *Bordetella pertussis* and *Klebsiella pneumonia* were found in one ETT each (4.35%).

**Figure 2:**
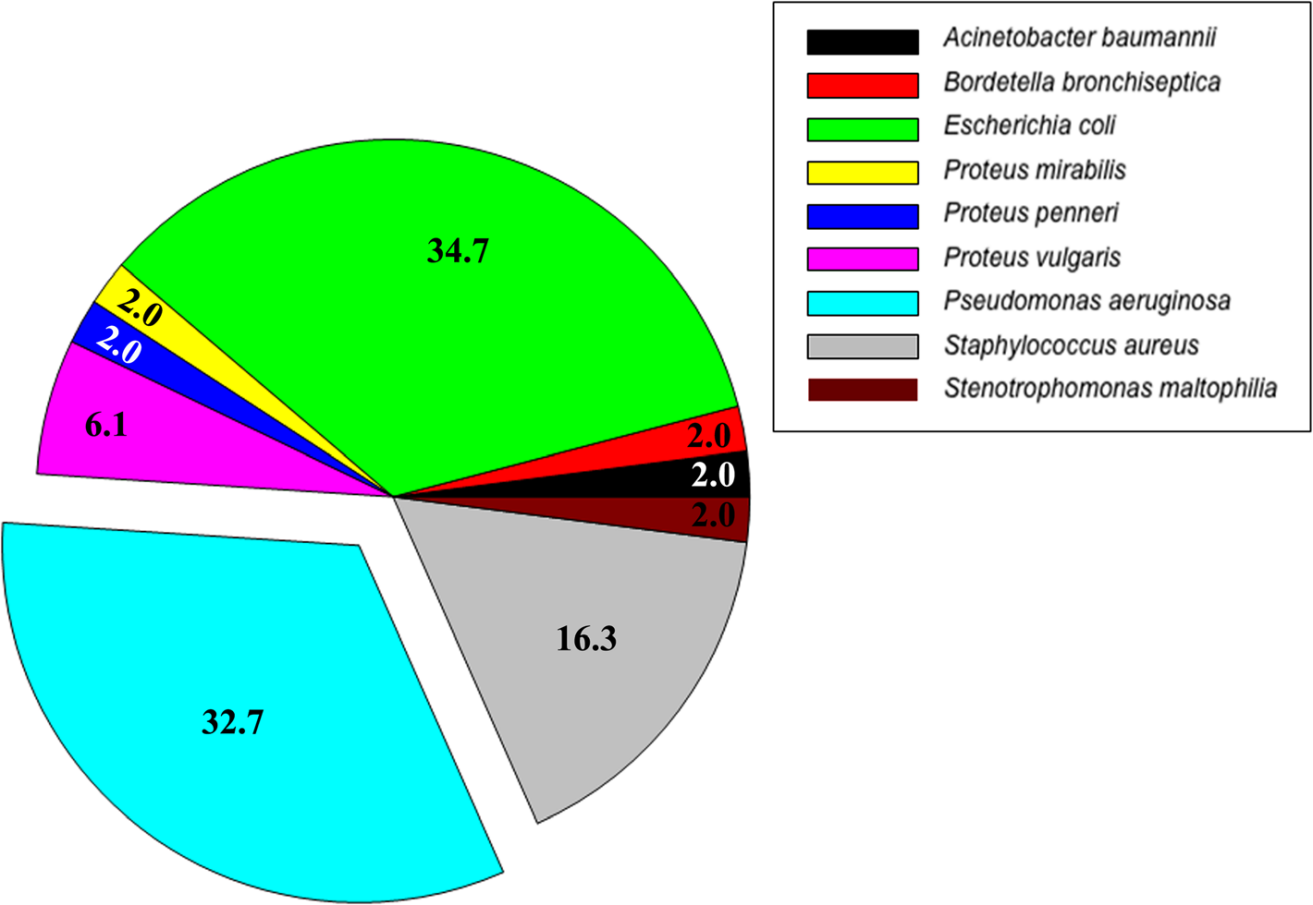
**Endotracheal tube colonization.** The pie chart represents the bacterial diversity recovered from the endotracheal tube upon extubation. The percentage of *Pseudomonas aeruginosa* isolates is explicitly indicated.

**Figure 3:**
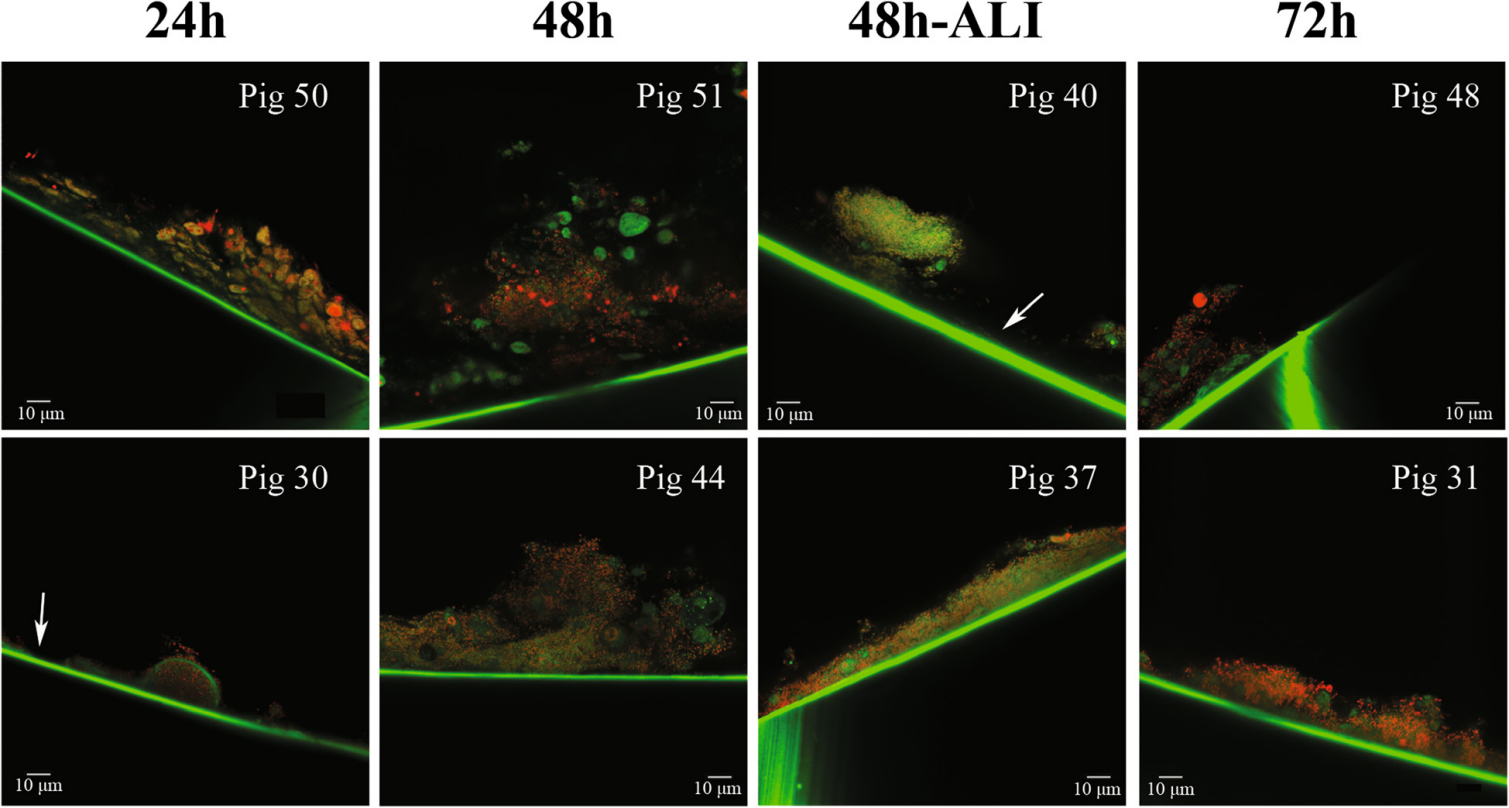
**Confocal laser scanning micrograph of the internal surface of endotracheal tubes of animals mechanically ventilated for 24, 48, 48 hours with concomitant lung injury and 72 hours.** The samples were stained with BacLight Live/Dead (magnification × 250). The white arrow indicates the endotracheal tube internal surface. Note in all pictures a fully mature biofilm adherent to the endotracheal tube.

**Figure 4:**
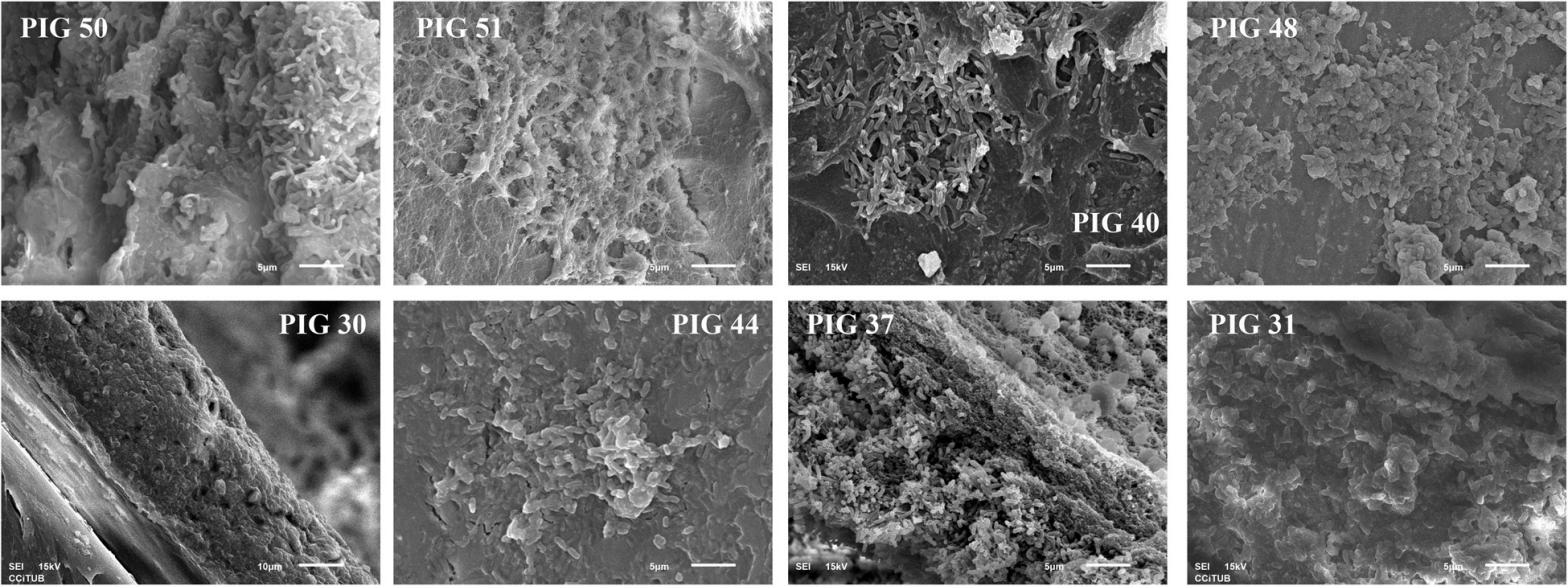
**Scanning electron micrographs of the internal surface of the endotracheal tube (ETT) in animals mechanically ventilated for 24, 48, 48 hours with concomitant lung injury and 72 hours.** Note in all pictures consistent presence of stage III/IV biofilm, characterized by multiple rod-shaped bacteria embedded within an extracellular polymeric substance. PIG 50, frontal view of the ETT lumen (magnification × 3,000); PIG 30, cross-section of the ETT lumen (magnification × 1,500); PIG 51, frontal view of the ETT lumen (magnification × 3,000); PIG 44, frontal-view of the ETT lumen (magnification × 3,000); PIG 40, frontal view of the ETT lumen (magnification × 3,000); PIG 37, frontal view of the ETT lumen (magnification × 3,000); PIG 48, frontal view of the ETT lumen (magnification × 3,000); PIG 31, frontal view of the ETT lumen (magnification × 3,000).

In the current study, upon extubation *P.aeruginosa* colonized the ETT internal lumen of 16 out of 18 ETTs (88.89%), at a mean concentration of 6.94 ± 0.77, 6.85 ± 1.14, 8.60 ± 0.69 and 8.21 ± 1.04 log cfu/mL in group 24 h, 48 h, 48 h-ALI and 72 h, respectively (*P* = 0.050). *P.aeruginosa* was not found in only one ETT of group 24 h, which was instead colonized by *Proteus vulgaris* (7.85 log cfu/mL) and *E.coli* (7.85 log cfu/mL); whereas, in one ETT of group 48-ALI the internal surface was only colonized by *Staphylococcus aureus* and *Bordetella bronchiseptica* at a concentration of 6.41 and 4.40 log cfu/mL, respectively. Among the ETTs colonized by *P.aeruginosa*, the number of days in storage was not associated with ETT colonization burden (N:16, r-square = 0.05, *P* = 0.386). In Figure 2 we report all pathogens cultured from the ETT inner lumen. Gram-negative pathogens accounted for 83.67% of the isolates, whereas 16.33% were Gram-positive. *S. aureus* were the most frequent Gram-positive bacteria. As clearly shown in Figures 3 and 4, stage III/IV biofilm was consistently present in all analyzed ETTs.

### Ventilatory management, airflows and pulmonary mechanics

Endotracheal suctioning was performed 5.2 ± 0.5, 4.5 ± 0.5, 5.0 ± 1.1, 4.3 ± 0.5 times per day in groups 24 h, 48 h, 48 h-ALI, 72 h, respectively (*P* = 0.100). As expected, in group 48 h-ALI, following oleic acid instillation the respiratory system elastance increased from 45.4 ± 4.0 to 75.0 ± 9.6 cm H_2_O/L (*P* = 0.002) and the ratio between arterial oxygen partial pressure and fraction of inspired oxygen (PaO_2_/F_I_O_2_) decreased from 454.0 ± 67.4 to 125.8 ± 61.1 (*P* <0.001). The respiratory rate in group 24 h was 19.8 ± 2.4 breaths per minute; 21.1 ± 1.9, in 48 h; 23.0 ± 1.9 (*P* = 0.026 versus 24 h, *P* = 0.008 versus 72 h) in 48 h-ALI and 20.8 ± 1.3 in 72 h. The tidal volume was 250.2 ± 17.6 mL and did not vary among groups. As a result, the inspiratory flow in group 24 h, 48 h, 48 h-ALI, 72 h was 16.7 ± 2.8, 19.1 ± 3.3, 21.5 ± 4.1 (*P* < 0.05 versus 24 h and 72 h) and 17.4 ± 3.0 L/min, respectively. The worst static respiratory system elastance was found in the 48-ALI group (58.6 ± 12.9 cm H_2_O/L, *P* <0.05 versus all groups) in comparison with 44.6 ± 7.2 in group 24 h, 46.8 ± 3.9 in 48 h and 48.9 ± 6.4 in group 72 h.

### Pulmonary colonization and infections

#### Tracheobronchial colonization by P. aeruginosa

**Figure 5:**
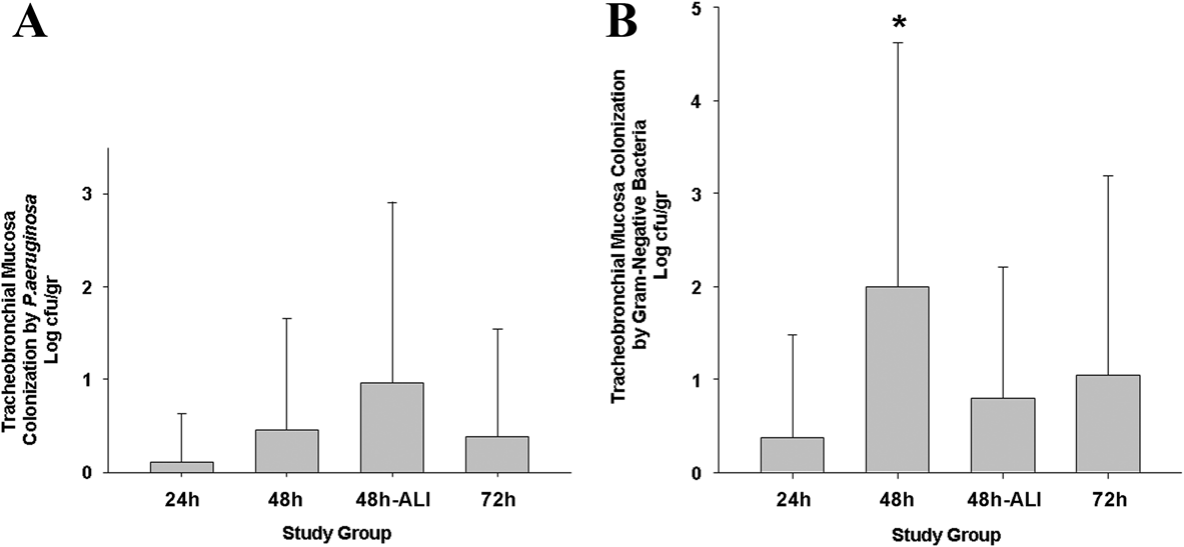
**Tracheobronchial mucosa bacterial concentrations for** ***P.aeruginos.*** **(A)** and all aerobic Gram-negative bacteria **(B)** per study group. The mean *P.aeruginosa* concentration did not differ among groups (*P* = 0.059), whereas, the concentration of all aerobic Gram-negative bacteria **(B)** was significantly different (*P* = 0.002). *P = 0.005 versus group 24 h. 24 h, 24 hours; 48 h, 48 hours; 48 h-ALI, 48 hours with concomitant acute lung injury; 72 h, 72 hours.

As reported in Table 1, colonization of the tracheobronchial tree by *P.aeruginosa* was found in 11 out of the 16 animals (68.7%) intubated with colonized ETTs. *P.aeruginosa* colonized 26 out of 198 samples (13.1%). In particular, 8.9% of the samples from the right lung were colonized, in comparison with 9.7% from the left lung (*P* = 0.856). *P.aeruginosa* mostly colonized the trachea and main bronchi (23.6%), and only 7.1% of the samples from the segmental bronchi (*P* = 0.001). *P.aeruginosa* was found in 4.5% of the samples of group 24 h, 12.7% of group 48 h, 21.8% of group 48 h-ALI and 11.4% of group 72 h (*P* = 0.085). The mean *P.aeruginosa* tracheobronchial concentration was 0.50 ± 1.38 log cfu/g and, as depicted in Figure 5A, only a trend toward higher colonization in group 48 h-ALI was found (*P* = 0.059). The mean colonization of the trachea and main bronchi was 0.97 ± 1.89 log cfu/g, in comparison with 0.23 ± 0.86 of the segmental bronchi (*P* <0.001). Post-hoc analyses confirmed that *P.aeruginosa* tracheobronchial concentration was not linearly associated with the length of stay on MV (*r*-square: 0.010, *P* = 0.222). Conversely, post-hoc analysis of animals ventilated for 48 h showed that *P.aeruginosa* tracheobronchial concentration was higher in animals with pulmonary injury, versus no injury, 0.95 ± 1.95 and 0.32 ± 1.03 log cfu/g, respectively (*P* = 0.016).

**Table 1:** **Tracheobronchial colonization (log cfu/g) per study group**

				**Trachea**		**Main bronchi**			**Segmental bronchi**					
**Pig**	**Group**	**Mechanical ventilation, hours**	**Species**	**1**	**2**	**3**	**4**	**5**	**6**	**7**	**8**	**9**	**10**	**11**
17	24 h	24	–	–	–	–	–	–	–	–	–	–	–	–
20	24 h	24	***P.aeruginosa***	2.18	2.74	–	–	–	–	–	–	–	–	–
			***E.coli***	1.70	2.30	–	–	–	–	–	–	–	–	–
30	24 h	24	*Bordetella bronchiseptica*	2.90	3.85	–	5.09	–	–	–	–	–	–	–
50	24 h	24	–	–	–	–	–	–	–	–	–	–	–	–
18	48 h	48	***P.aeruginosa***	3.69	3.95	4.62	2.40	–	–	2.96	–	–	–	–
27	48 h	48	*Bordetella bronchiseptica*	6.19	7.24	7.14	7.37	5.76	5.16	5.99	–	3.70	3.66	–
44	48 h	48	***P.aeruginosa***	–	–	–	–	–	–	2.95	–	–	–	–
			*Bordetella bronchiseptica*	–	3.60	–	3.86	3.83	–	3.02	–	–	–	–
			***E.coli***	–	–	–	–	–	–	2.88	–	–	–	–
			*Acinetobacter baumanii*	–	–	–	–	–	–	3.08	–	–	–	–
51	48 h	48	***P.aeruginosa***	–	–	–	4.05	–	–	–	–	–	–	–
55	48 h	48	*Bordetella bronchiseptica*	5.56	4.60	5.33	7.12	5.76	4.63	5.72	2.30	–	–	–
34	48-ALI	48	***P.aeruginosa***	4.68	5.02	–	6.41	–	–	–	4.24	4.18	–	3.85
			***E.coli***	4.08	–	–	–	–	–	–	–	–	–	–
37	48-ALI	48	***P.aeruginosa***	1.78	–	–	–	–	–	–	–	–	–	–
			*Acinetobacter lwoffii*	–	–	–	–	–	–	–	–	–	–	2.17
40	48-ALI	48	***P.aeruginosa***	–	–	3.21	–	–	–	–	–	–	–	–
52	48-ALI	48	***P.aeruginosa***	6.86	2.95	–	5.86	–	–	–	–	3.76	–	–
			*E.coli*	4.54	2.52	–	–	–	–	–	–	–	–	–
54	48-ALI	18	***Bordetella bronchiseptica***	3.80	3.12	3.23	3.32	2.18	3.09	3.11	2.40	3.26	–	2.75
22	72 h	72	***P.aeruginosa***	4.64	–	–	–	–	–	–	–	–	–	–
			*Bordetella bronchiseptica*	6.60	5.30	–	5.35	5.72	–	–	–	6.40	–	–
			*Paracoccus yeei*	–	–	–	–	–	–	–	–	–	5.60	–
31	72 h	72	–	–	–	–	–	–	–	–	–	–	–	–
48	72 h	72	***P.aeruginosa***	–	–	–	–	1.77	–	–	–	–	–	–
53	72 h	72	***P.aeruginosa***	4.86	–	–	–	3.45	2.08	–	–	–	–	–
			***E.coli***	4.18	–	–	–	3.79	2.94	–	–	–	–	–

Of note, bacterial species concurrently isolated from within the endotracheal tube and the tracheobronchial tree are in bold. *P.aeruginosa, Pseudomonas aeruginosa; E.coli, Escherichia coli.*

#### Tracheobronchial colonization by other aerobic Gram-negative bacteria

*B. bronchiseptica* was the most frequent pathogen, isolated in 75% of the colonized samples; *E. coli* was found in 19.2% samples, whereas *Acinetobacter lwoffi, Acinetobacter baumanii* and *Paracoccus yeii* were each isolated in 1.9% of the samples. *E. coli* colonized both the ETT and the tracheobronchial tree in five animals; whereas *B. bronchiseptica* only in one animal. Tracheal and main bronchi samples were more frequently colonized (36.1% of the samples), in comparison with only 18.2% of the segmental bronchi samples (*P* = 0.005). In group 48 h, 38.2% of the samples were colonized versus 11.4% in group 24 h, and 20.4% in group 72 h (*P* = 0.018). As reported in Figure 5B, the highest concentration of other aerobic Gram-negative bacteria was found in group 48 h.

#### Pulmonary infections

Data on pulmonary infections are depicted in Table 2. Upon study completion respiratory infections were clinically suspected in 13 out of 18 animals (72.2%). Incidence of clinically suspected pulmonary infections did not vary among groups. *P.aeruginosa* VAT was microbiologically confirmed in 5 out of 13 animals (38.5%). Animals of group 24 h never developed respiratory infections, whereas 60% of the animals in group 48-ALI developed *P.aeruginosa* VAT. Among the animals with clinical suspicion of pulmonary infections, the right and left upper lobes were the sites most commonly sampled because of apparent gross findings of infection. Nevertheless, *P.aeruginosa* VAP was never microbiologically confirmed. As for pulmonary infections caused by other Gram-negative bacteria, VAT was microbiologically confirmed in five animals, mostly of group 48 h-ALI (three out of five animals (60%)). VAP by other Gram-negative pathogens never developed. VAT was caused by *B. bronchiseptica* in 66.6% of the cases and *E. coli* in the remaining cases. In 50% of the cases of VAT, there was a concomitant isolation of *P.aeruginosa* and another Gram-negative pathogen.

**Table 2:** **Pulmonary infections per study group**

							***Pseudomonas Aeruginosa***		**Gram-negative bacteria**		
**Pig**	**Group**	**Hours of MV**	Fever* (°C)	WBC* (cells/mm3)	Purulent secretions*	**Clinical suspicion of VAP/VAT**	**Microbiologically confirmed VAP**	**Microbiologically confirmed VAT**	**Microbiologically confirmed VAP**	**Species**	**Microbiologically confirmed VAT**
17	24 h	24	37.6	21400	No	No	No	No	No	–	No
20	24 h	24	40.2	23100	No	**Yes**	No	No	No	–	No
30	24 h	24	38.5	19700	No	**Yes**	No	No	No	–	No
50	24 h	24	37.4	39500	Yes	**Yes**	No	No	No	–	No
18	48 h	48	39.2	20500	No	**Yes**	No	**Yes**	No	–	No
27	48 h	48	38.3	28000	No	No	No	No	No	–	No
44	48 h	48	36.2	21700	Yes	**Yes**	No	No	No	*Bordetella bronchiseptica*	**Yes**
51	48 h	48	37.6	18200	No	No	No	No	No	–	No
55	48 h	48	36.1	18300	Yes	**Yes**	No	No	No	*Bordetella bronchiseptica*	**Yes**
34	48-ALI	48	36.0	16400	Yes	**Yes**	No	**Yes**	No	*Escherichia coli*	**Yes**
37	48-ALI	48	NA	21600	Yes	**Yes**	No	No	No	–	No
40	48-ALI	48	38.9	29000	Yes	**Yes**	No	**Yes**	No	–	No
52	48-ALI	48	37.6	24900	Yes	**Yes**	No	**Yes**	No	*Escherichia coli*	**Yes**
54	48-ALI	18	NA	24700	Yes	**Yes**	No	No	No	*Bordetella bronchiseptica*	**Yes**
22	72 h	72	NA	18600	Yes	**Yes**	No	**Yes**	No	*Bordetella bronchiseptica*	**Yes**
31	72 h	72	36.0	22000	Yes	**Yes**	No	No	No	–	No
48	72 h	72	37.0	22700	No	No	No	No	No	–	No
53	72 h	72	36.5	21700	No	No	No	No	No	–	No

Pulmonary infections were clinically suspected in the case of at least two of three of the following clinical features: body temperature >38.5°C or <36°C; white blood count >14,000/mm^3^ or <4,000/mm^3^ and purulent secretions. VAT was microbiologically confirmed when a bronchial mucosa culture was ≥3 log cfu/g. VAP was microbiologically confirmed according to a lung tissue quantitative bacterial culture ≥3 log cfu/g. Of note, only pulmonary lobes with apparent gross findings of pneumonia (edematous tissue; consolidated areas; mucopurulent material in the tracheobronchial tree; abscess) were sampled, whereas in group 48-ALI all lobes were sampled. *Only values upon autopsy are reported in the table, but statistical analysis included all values collected every 12 hours: *P*-values for differences among groups were 0.212 (Fever), 0.276 (WBC), 0.298 (Purulent secretions), 0.439 (Clinical suspicion of VAP/VAT), not significant (Microbiologically confirmed VAP) and 0.327 (Microbiologically confirmed VAT) for *P. Aeruginosa*, and not significant (Microbiologically confirmed VAP) and 0.423 (Microbiologically confirmed VAT) for Gram-negative bacteria. ALI, acute lung injury; MV, mechanical ventilation; VAP, ventilator-associated pneumonia; VAT, ventilator associated tracheobronchitis; WBC, white blood cell; 24 h, 24 hours; 48 h, 48 hours; 48 h-ALI, 48 hours with concomitant acute lung injury; 72 h, 72 hours.

## Discussion

This laboratory animal study demonstrates that following intubation with an ETT colonized by mature *P.aeruginosa* biofilm, there is a consistent translocation of pathogens into the airways. Nevertheless, in animals on MV up to 72 hours and in LTP, the ETT biofilm only colonizes the proximal airways and most importantly it does not cause VAP, even in injured lungs.

During MV, biofilm rapidly forms on the internal surface of ETTs [12,27]. ETT biofilm is a complex structure made of pathogens enclosed within a self-produced polymeric matrix, and respiratory secretions. The accumulation of biofilm and secretions within the ETT progressively obstructs its lumen, particularly in patients on long-term MV [28,29]. This increases the patient work of breathing and potentially delays liberation from MV [30]. The inspiratory flow interacts with the biofilm surface [31], which become unstable and eventually particles may be disseminated into the airways [12]. Additionally, ETT suctioning [32] or other invasive respiratory procedures, that is, bronchoscopy, may dislodge clumps of pathogens and biofilm elements. Antibiotics do not eradicate ETT biofilm [33,34], and only dedicated devices have shown full removal of biofilm and secretions from within the ETT [35-37]. ETT biofilm plays a role in the development of VAP. Indeed, upon extubation in patients with VAP the internal ETT lumen is fully covered by airway secretions and pathogens within a thick biofilm [12,15,37]. Importantly these pathogens often match causative VAP microorganisms [12]. Therefore, several investigators have suggested that ETT biofilm might play a role in the development of VAP. To the best of our knowledge this is the first report that challenges this theory; indeed, we demonstrated that in pigs placed in the LTP, ETT biofilm is mostly associated with VAT rather than VAP.

In clinical settings, several preemptive interventions are applied to prevent VAT and VAP. Among those strategies international guidelines [38,39] strongly recommend that enterally fed patients on MV be kept in the semirecumbent position to prevent reflux of colonized gastric contents and the gastro-pulmonary route of colonization [1]. Yet results from laboratory studies [3-6] in large animals challenge the use of the semirecumbent position in patients with oropharyngeal colonization, because bacteria may be driven into the airways through gravity. In particular, studies in tracheally intubated pigs [4,5] and sheep [4] on MV for up to 168 hours report an incidence of VAP close to 95% in animals in the semirecumbent position. Conversely, mucus clearance was drastically improved and pulmonary infections fully prevented in animals positioned in the LTP.

In our study, we used ETTs already colonized by *P.aeruginosa* at the time of intubation, and the LTP. Therefore, it is rational to assume that *P.aeruginosa* disseminated only from within the ETT biofilm, and the concomitant inoculation of endogenous oropharyngeal pathogens was limited because of the position and prophylactic antibiotic therapy with ceftriaxone. Importantly, colonized ETTs were obtained from a previous study [3] in which animals underwent MV for 72 hours and presented oropharyngeal colonization by *P.aeruginosa*. Thus, in the current study, we used ETTs with late-stage biofilm formation. Interestingly, colonization by *P.aeruginosa* was not corroborated in 2 out of 18 ETTs. This was likely related to the bacterial diversity and complex ecology of the studied ETTs and the vast presence of microbial competitors, that is, *P. vulgaris, B. bronchiseptica, E.coli* and *S. aureus.* In a previous canine model of ETT biofilm-associated pulmonary infection [17], the investigators challenged the oropharynx with *P.aeruginosa*. This led to aspiration of colonized secretion into the airways, extensive biofilm formation within the ETT and ultimately to VAP. Although this model closely simulated the pathogenic mechanisms of VAP in clinical settings, it was impossible to differentiate between the role of ETT and oropharyngeal colonization in the development of the infection. Consequently, this is the first study that substantiates a preventive effect of the LTP in the development of pulmonary infections specifically caused by ETT biofilm. Other important features of our model were the complete absence of PEEP [40] and the development of lung injury [41,42] in one of the groups. These settings were specifically employed to replicate the worst possible clinical conditions for the development of respiratory infections during MV. According to previous studies [17] extensive biofilm formation is found in all ETTs. Nevertheless, we also found a trend toward higher ETT colonization in group 48-ALI and 72-h. These animals were at greater risk of VAP because of lung injury and prolonged time on MV; thus, this imbalance between groups further validates the benefits of the LTP. Yet we found slightly enhanced pulmonary colonization in group 48-ALI. This could be explained by the impairment of the host immune response, due to the lung injury and the ventilatory settings. Indeed, in these animals the respiratory rate was greater to maintain normocapnia, and as a result higher inspiratory airflow was generated. This could have led to flow turbulence within the ETT and substantial dislodgement of biofilm particles.

This study has a few limitations that need to be highlighted. First, ETT colonized by *P.aeruginosa* were employed, hence our results may not be valid for other bacterial species. Second, although we did not find a progressive increase in risk of pulmonary colonization associated with the time on MV, the animals were ventilated for up to 72 hours and our results may not be relevant during prolonged time on MV. Third, we found a concomitant colonization by endogenous pathogens, namely, *B. bronchiseptica* and *E. coli* in a few animals. This could have caused active competition for colonization, and led to decreased risk of *P.aeruginosa* colonization. Additionally, in the current and the associated study, microbiology samples of the oropharynx and gastric contents were not collected, thus, the specific origin of bacteria colonizing the ETTs remains uncertain. Fourth, in the present study, we did not test whether the semirecumbent position could further promote biofilm-associated pulmonary infections. Yet several of our previous studies [3,6,18] confirmed that such a position consistently caused pulmonary infections through gravity-driven aspiration of oropharyngeal contents, and likely through translocation of ETT pathogens. Last, although sample size analysis indicated that three animals per group would be sufficient to demonstrate our goals, larger laboratory and clinical studies should validate our results. Nevertheless, it is important to emphasize that we did not find any trend toward an increased risk of VAP associated with the length of stay on MV and lung injury.

## Conclusions

In summary, this animal study implies that during the course of invasive MV, *P.aeruginosa* biofilm within the ETT rapidly colonizes the proximal respiratory tract. Nevertheless, when animals are positioned in the LTP, bacterial colonization is compartmentalized within the proximal airways and during the first 72 hours of MV plays a marginal role in the development of VAP.

## Key messages

- Following intubation with an endotracheal tube colonized by mature *P. aeruginosa* biofilm, there is a consistent translocation of pathogens into the airways.
- In animals positioned in the lateral Trendelenburg position and mechanically ventilated for up to 72 hours, endotracheal tube *P. aeruginosa* biofilm mostly colonizes the trachea and main bronchi
- However, in the lateral Trendelenburg position, endotracheal tube *P. aeruginosa* biofilm only causes ventilator-associated tracheobronchitis.
- Lung injury marginally increases risks of endotracheal tube *P. aeruginosa* biofilm pulmonary colonization and infection.

## Abbreviations

ALI: acute lung injury
cfu/g: colony forming units per gram
CSLM: confocal scanning laser microscopy
ETT: endotracheal tube
LTP: lateral-Trendelenburg position
MV: mechanical ventilation
PEEP: positive end-expiratory pressure
SEM: scanning electron microscopy
VAP: ventilator-associated pneumonia
VAT: ventilator-associated tracheobronchitis
